# Effects of pharmacogenomics-guided treatment on medication adherence and the antidepressant switching rate in major depressive disorder

**DOI:** 10.3389/fphar.2024.1501381

**Published:** 2024-11-29

**Authors:** Chaoli Chen, Yang Lun, Jing Yu, Xiaochuan Zhao, Shi Su, Mengqiang Zhao, Yuhang Yan, Jiaqi Wang, Ran Fu, Feiyue An, Liguang Duan, Leting Yan, Ruxing Li, Jinxiao Li, Ziyu Liu, Xiaoying Geng, Jincheng Wang, Yuanyuan Zhao, Chunhua Zhou

**Affiliations:** ^1^ Department of Clinical Pharmacy, The First Hospital of Hebei Medical University, Shijiazhuang, China; ^2^ Department of the Technology Innovation Center for Artificial Intelligence in Clinical Pharmacy of Hebei Province, Shijiazhuang, China; ^3^ Department of Psychiatry, The First Hospital of Hebei Medical University, Shijiazhuang, China; ^4^ Key Laboratory for Neuroimmunological Regulation and Mental Health of Hebei Province, Shijiazhuang, China; ^5^ Pharmacy Department, The First Hospital of Hebei Medical University, Shijiazhuang, China; ^6^ School of Pharmacy, Hebei Medical University, Shijiazhuang, China; ^7^ Management Department, The First Hospital of Hebei Medical University, Shijiazhuang, China

**Keywords:** pharmacogenomics, adherence, switching, depression, antidepressant

## Abstract

**Background:**

In the treatment of depression, medication plays a crucial role. However, insufficient patient adherence to medication often results in unsatisfactory treatment outcomes, increasing both the recurrence and rehospitalization rates of depression, and consequently imposing a greater economic burden on the healthcare system.

**Objectives:**

Our objective was to examine the impact of pharmacogenomic testing on medication adherence and antidepressant switching rates among individuals diagnosed with depression.

**Methods:**

This retrospective cohort study encompassed patients diagnosed with depression who were admitted to the First Hospital of Hebei Medical University between April 2022 and September 2023. Patients were categorized into a pharmacogenomics-guided treatment (PGxT) group and a treatment as usual (TAU) group based on whether pharmacogenetic testing was conducted. The primary outcome measures included the proportion of patients exhibiting medication adherence greater than 80% at three and 6 months post-discharge, as well as the proportion of patients experiencing changes in their prescribed medication types.

**Results:**

A total of 310 patients in the PGxT group and TAU group were obtained through propensity score matching. Among the 620 patients in both groups, 57.42% demonstrated good adherence (≥80%) at 3 months; this percentage dropped to 31.45% at 6 months. At 3 months of observation, the percentages of patients demonstrating good adherence were significantly different between the groups (64.52% in the PGxT group vs. 50.32% in the TAU group; *p* < 0.001). The difference was also significant after 6 months (38.06% in the PGxT group vs. 24.84% in the TAU group; *p* < 0.001). Furthermore, patients receiving PGxT (20.64%) exhibited a lower rate of antidepressant conversion compared to those receiving TAU (31.29%).

**Conclusion:**

The findings of this study indicate that pharmacogenomics testing positively influences treatment adherence and may decrease the need to switch medications among patients with depression.

## Introduction

Worldwide, the incidence of major depressive disorder (MDD) is increasing annually, resulting in greater economic and social burdens (Cui et al., 2024). The World Health Organization identifies it as one of the leading causes of disability worldwide (World Health Organization, 2017). Symptoms of depression can include severe mood decline, insomnia, appetite fluctuations, and reduced physical stamina, which severely impact quality of life and work capacity (Cui, 2015). Addressing depression involves a multifaceted, long-term strategy. Treatment modalities include medication and psychotherapy, often supplemented by additional therapies such as exercise and music therapy. Among these, pharmacotherapy is particularly critical. According to the 2023 guidelines of the Réseau Canadien pour les Traitements de l’Humeur et de l’Anxiété (CANMAT), for major depressive episodes of moderate severity and low-to-moderate safety risk, the initial treatment options should be either structured psychotherapy or antidepressant medication, as these modalities demonstrate the most robust and consistent evidence of safety and efficacy. In the cases of severe major depressive episodes without psychotic features but with a high safety risk, CANMAT recommends combining antidepressant medication with psychotherapy (Lam et al., 2024). However, medication adherence remains low among patients, often due to the lack of understanding of depression by patients and their families, poor patient self-awareness, and adverse drug reactions. Additionally, physicians usually choose antidepressants based on their clinical experience, which can neglect individual patient differences. This oversight can lead to suboptimal drug efficacy or adverse reactions, thus reducing patient adherence and compromising therapeutic outcomes (Ho et al., 2017).

Poor adherence to medication leads to suboptimal treatment outcomes, including increased rates of relapse and hospital readmission, thus exacerbating the financial burden on healthcare systems (Ho et al., 2016). Analyses of extensive Medicaid databases reveal that patients who fill their antidepressant prescriptions at the initial visit and maintain adherence to at least two-thirds of the prescribed regimen over the following 6 months are less prone to relapses and depressive episodes. In contrast, abrupt cessation of antidepressants is associated with a 77% higher risk of relapse or recurrence (Melfi et al., 1998). Furthermore, high non-response rates to antidepressants, ranging from 30% to 50%, pose a significant clinical challenge (Bschor et al., 2014). A systematic review and meta-analysis focused on the efficacy of switching antidepressants after medication non-response in adults with major depressive disorder did not find high-level evidence to support the superiority of switching medications over continuing with the initial treatment (Bschor et al., 2018). Therefore, identifying effective treatment strategies after the failure of antidepressant monotherapy remains a critical clinical challenge in the management of depression. Although switching antidepressants is common in clinical practice, controlled trials that provide evidence of the efficacy of this approach are still lacking.

Pharmacogenomics (PGx) is an area of science that examines the role of inherited genetic differences as a determinant of interpatient variation in drug disposition and response (Medwid and Kim, 2024). Pharmacogenomics (PGx) has been increasingly utilized to optimize the selection of antidepressant medications. Pharmacogenomics studies the effects of genes and genetic variation on how an individual responds to certain medications or combinations of medications (Virelli et al., 2021). By detecting these variations, pharmacogenomic testing can provide valuable information for medication selection and dose adjustment in clinical practice (Kang et al., 2023). A systematic review of studies assessing the cost-effectiveness of pharmacogenetic testing reported that 71% of the studies found the testing to be cost-effective or even cost-saving (Morris et al., 2022). This suggests that PGxT could reduce healthcare expenditures and enhance patient compliance. Additionally, the estimated probabilities of receiving an antidepressant with no, moderate, and substantial drug-gene interactions after PGxT were 59.3%, 30.0%, and 10.7%, respectively, compared to 25.7%, 54.6%, and 19.7%, under treatment as usual (TAU) (Oslin et al., 2022). This indicates that pharmacogenomics-guided treatment (PGxT) significantly reduces patients’ likelihood of receiving medications with potential adverse effects. Finally, a systematic review and meta-analysis of prospective, controlled clinical studies revealed a modest but significant positive impact of PGxT on the remission of depressive symptoms. Individuals receiving PGxT were 41% more likely to achieve remission than those under TAU, with a 95% confidence interval of 15%–74% (Brown et al., 2022). These findings show the potential of PGxT to benefit symptom remission, particularly during long-term treatment significantly. Research by Koufaki et al. found that *P*Gx was considered to promote patient-centered care, enhance medication clinical effectiveness, decrease the risk of side effects, and reduce healthcare costs (Koufaki et al., 2024).

The primary objective of this study was to compare the effectiveness of PGxT with TAU in managing major depressive disorder. The comparison was centered on two key outcomes, including medication adherence, and frequency of antidepressant switching due to drug inefficacy or side effects. By focusing on these outcomes, we aimed to provide a comprehensive understanding of the potential benefits of PGxT in the management of major depressive disorder.

## Materials and methods

### Participants and study design

This retrospective cohort study involved patients hospitalized at the First Hospital of Hebei Medical University from April 2022 to September 2023. Data on medication use were collected from the time of discharge up to 6 months after discharge. Inclusion criteria included: (1) a diagnosis of depression according to the International Classification of Diseases (ICD), Tenth Edition; (2) a score of 20 or higher on the 24-item Hamilton Depression Scale (HAMD), and (3) treatment with at least one antidepressant medication. The exclusion criteria were: (1) a diagnosis of bipolar disorder, psychotic disorders with hallucinations or delusions, schizophrenia, or generalized anxiety disorder; (2) absence of at least one outpatient follow-up visit to our hospital after discharge; and (3) readmission to the hospital during the 6-month observation period.

In this study, we strictly adhered to the principles of data privacy and confidentiality. All patient data were rigorously de-identified prior to analysis. The study protocol was approved by the Clinical Research Ethics Committee of the First Hospital of Hebei Medical University (approval number: [2024] Research Review No. 146).

### Logic of gene detection and interpretation of pharmacogenomics

In the psychiatric department of the First Hospital of Hebei Medical University, 33 loci in 8 genes (Supplementary Table S1) were selected for PGxT of 14 commonly used antidepressant drugs based on clinical guidelines and recommendations from the PharmGKB database (https://www.Pharmgkb.org). These genes predominantly include metabolism (CYP2D6, CYP2C19, CYP2B6, CYP1A2) and efficacy (ABCB1, HTR1A, HTR2A, SLC6A4). We performed an in-depth analysis of each candidate site, as detailed in (Supplementary Tables S2, S3), and summarized the phenotype and evidence levels for the 14 drugs across these genes in (Supplementary Table S4), with evidence-level classifications drawn from the PharmGKB database and the pharmacogenomics guidelines of the China National Health and Family Planning Commission (Hicks et al.,2015; Benowitz et al., 2013; Lin et al., 2010; Saiz-Rodríguez et al., 2019; Jeleń et al., 2015; Uhr et al., 2008; Kato et al., 2009; Tiwari et al., 2013; Uher et al., 2009; Zanardi et al., 2001; Popp et al., 2006).

We used nucleic acid mass spectrometry technology to establish a set of pharmacogenomics (PGx) detection methods for the relevant genetic loci, detailed in **(** Supplementary Method **)**. Following the characterization of the 8-gene panel and associated antidepressants, we formulated interpretation principles for the drug recommendations (Supplementary Figure S1). Depending on each patient’s genotype, we recommended one of three prescription categories for each drug: “standard prescription,” “use with caution,” or “adjustment needed,” as informed by prior research (Hall-Flavin et al., 2013). As shown in (Supplementary Figure S2; Supplementary Table S4), the gene variants associated with escitalopram include CYP2C19, SLC6A4, and HTR2A, with evidence levels of 1A, 4, and 3, respectively, according to the PharmGKB database. Recommendations are determined by the metabolic type of CYP2C19: ultra-rapid metabolizer (UM) or poor metabolizer (PM) immediately warrants an “adjustment needed” designation. If the patient is an extensive metabolizer (EM) or intermediate metabolizer (IM), further assessment involving the SLC6A4 and HTR2A genes is required; an SLC6A4 genotype of L/L or L/S and an HTR2A genotype of CC or CG lead to a “standard prescription,” while an SLC6A4 genotype of S/S or an HTR2A genotype of GG triggers a “use with caution” recommendation.

Before undergoing PGxT, all participants provided their informed consent. The results of the tests were documented in each patient’s electronic health record, ensuring that the treating physicians had timely access to the information. All physicians involved received professional training to facilitate accurate interpretation of pharmacogenetic results and enable appropriate clinical decision-making. This training was designed to enhance their proficiency in understanding and applying the information contained in the pharmacogenetic reports.

### Assessment of medication adherence

The proportion of days covered (PDC) is a quantitative metric to assess medication adherence. The PDC is calculated as the ratio of the number of days a patient takes the prescribed medication to the total number of days in the observation period, with a PDC of 80% or higher is considered indicative of good adherence (Benner et al., 2002). This study assessed adherence by calculating the PDC for outpatient prescriptions. A modified PDC (mPDC) was used, as described by Christian et al. (Christian et al., 2020), to accommodate patients who might change antidepressants during the observation period due to treatment ineffectiveness or adverse reactions. Unlike standard PDC, the mPDC calculation did not adjust for the number of medications when a patient switched antidepressants, considering both the type and quantity of medications used.

$$\text{number of days covered}=\frac{\text{meds}\;\text{dispensed}*}{\text{number}\;\text{of}\;\text{times}\;\text{taken}\;\text{daily}}$$

$${\text{mPDC}}_{\text{Drug}}=\frac{\sum \left(\text{number}\;\text{of}\;\text{days}\;\text{coverd}\right)+\sum \left(\text{days}\;\text{covered}\;\text{from}\;\text{previous}\;\text{pre}s\text{cription}\;\text{during}\;\text{gap}\;\text{periods}\right)}{\text{last}\;\text{end}\;\text{order}\;\text{time}-\text{index}\;\text{start}\;\text{order}\;\text{time}}\times 100\%$$

$${\text{mPDC}}_{\text{Overall}}=\frac{{\text{PDC}}_{\text{Drug}1}+{\text{PDC}}_{\text{Drug}2}+\cdots +{\text{PDC}}_{\text{DrugN}}}{\text{number}\;\text{of}\;\text{drugs}}$$

or

$${\text{mPDC}}_{\text{Overall}}={\text{PDC}}_{\text{Drug}1}+{\text{PDC}}_{\text{Drug}2}+\cdots +{\text{PDC}}_{\text{DrugN}}$$

* If the number of medications dispensed exceeded the duration of its corresponding electronic prescription order, and a subsequent prescription for the same medication was electronically prescribed, the number of medications dispensed was adjusted to match the duration of the electronic prescription order. Additionally, any remaining medications from a previous order were factored into the mPDC calculation for the intervals between electronic prescription orders.

### Evaluation of the antidepressant switching rate

Adjusting medication types is a crucial indicator to assess medication effectiveness in clinical practice, with changes in medication reflecting treatment responsiveness and efficacy to some extent. In this study, the antidepressant switching rate (ASR) was used to gauge the responsiveness and efficacy of antidepressant treatment. ASR was calculated as the ratio of patients who switched antidepressants during treatment to the total number of patients in each group.

$$\text{ASR}=\frac{\text{Number}\;\text{of}\;\text{patients}\;\text{switching}\;\text{antidepressants}*}{\text{Total}\;\text{number}\;\text{of}\;\text{observations}}$$

* A patient was considered to have switched antidepressants if there was a change in medication type from the time of hospital discharge until the end of the observation period.

### Statistical analysis

Patient descriptive statistics are presented as the median and interquartile range (IQR) for continuous variables and frequency and percentage for categorical variables. For continuous data, statistical comparisons were conducted using the Mann-Whitney *U* test. Categorical data were analyzed using Pearson’s chi-square test or Fisher’s exact test, as appropriate. *P* < 0.05 were considered statistically significant. All statistical analyses were performed with SPSS version 27.

## Results

### Patient characteristics

**FIGURE 1:**
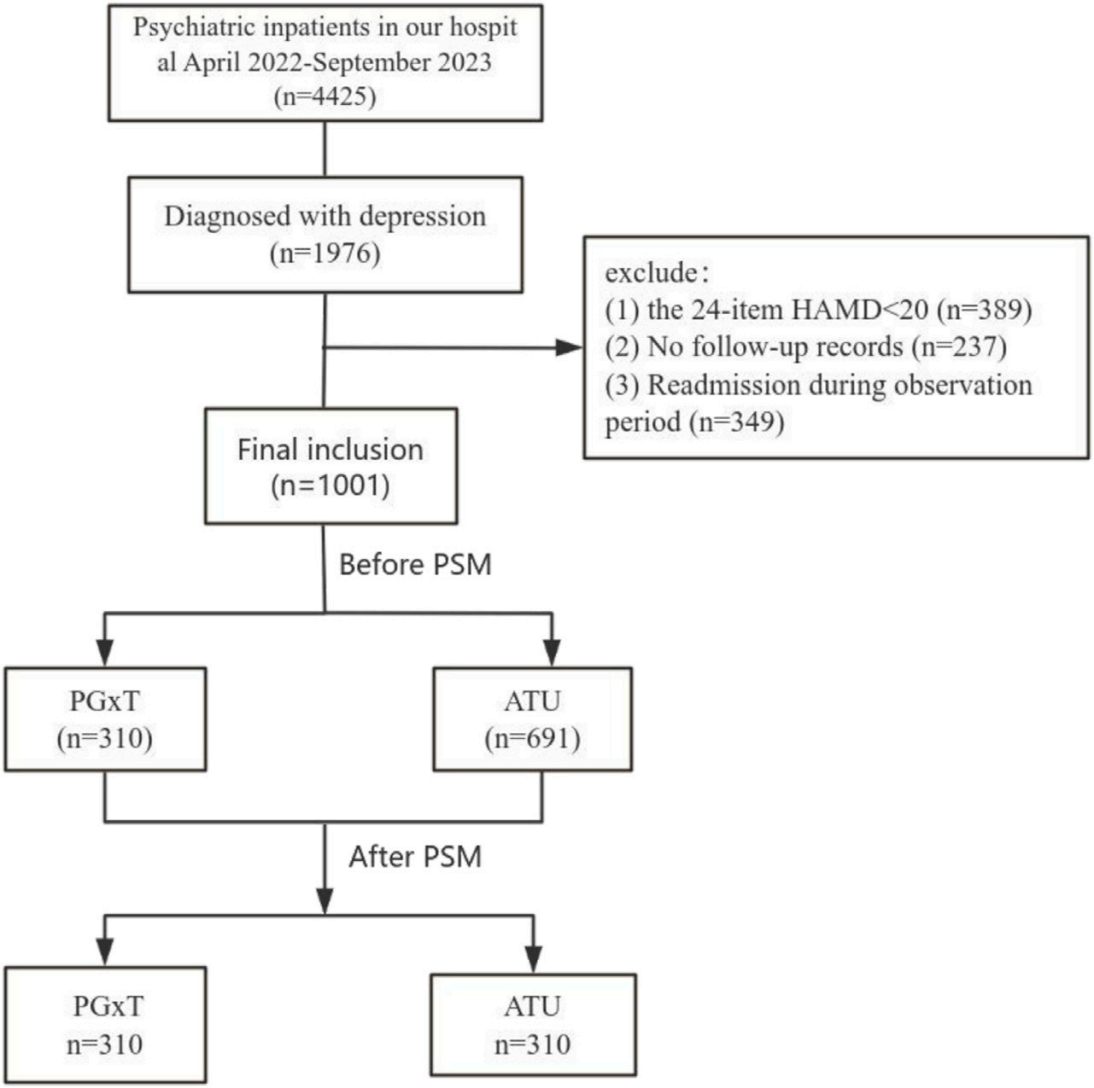
Flow chart of patient inclusion.

We reviewed the medical records of patients hospitalized in the psychiatric department of the First Hospital of Hebei Medical University from April 2022 to September 2023. Initially, 4,425 patients were identified, with 3,424 excluded for not meeting the inclusion and exclusion criteria. The reasons for exclusion included: (1) diagnosis of bipolar disorder, psychotic disorders with hallucinations or delusions, schizophrenia, or generalized anxiety disorder (n = 2,449); (2) 24-item HAMD scores below 20 (n = 389); (3) absence of follow-up in an outpatient clinic after discharge (n = 237); and (4) rehospitalization within 6 months after discharge (n = 349). Ultimately, 1,001 patients qualified for the study and were divided into the PGxT group (n = 310) and the TAU group (n = 691). No significant differences were observed in the gender ratios and HAMD scores between the groups. However, significant differences in age distributions were observed. To ensure data comparability between groups, a logistic regression model was employed to calculate each subject’s propensity score matching (PSM) using gender, age, and HAMD score as covariates. PSM resulted in 310 successfully matched pairs (totaling 620 cases; Figure 1). Table 1 shows the baseline characteristics of the data before and after proximity matching.

**TABLE 1:** Summary of baseline population characteristics.

Characteristics	Before PSM			After PSM		
	PGxT (*n* = 310)	TAU (*n* = 691)	*p*	PGxT (*n* = 310)	TAU (*n* = 310)	*p*
Age, median (IQR)	50.00 (23.75,60.00)	55.00 (30.00,66.00)	0.001	50.00 (23.75,60.00)	49.00 (20.00,62.00)	0.958
sex			0.562			0.607
Male (%)	104 (33.5%)	219 (31.7%)		104 (33.5%)	98 (31.6%)	
Female (%)	206 (66.5%)	472 (68.3%)		206 (66.5%)	212 (68.4%)	
HAMD, median (IQR)	39.00 (28.00,49.25)	38.00 (31.00,45.00)	0.051	39.00 (28.00,49.25)	39.00 (31.00,47.00)	0.396

IQR, interquartile range; HAMD, hamilton depression scale; PSM, propensity score matching; PGxT, pharmacogenomics-guided treatment; TAU, treatment as usual.

### Comparison of medication adherence

**FIGURE 2:**
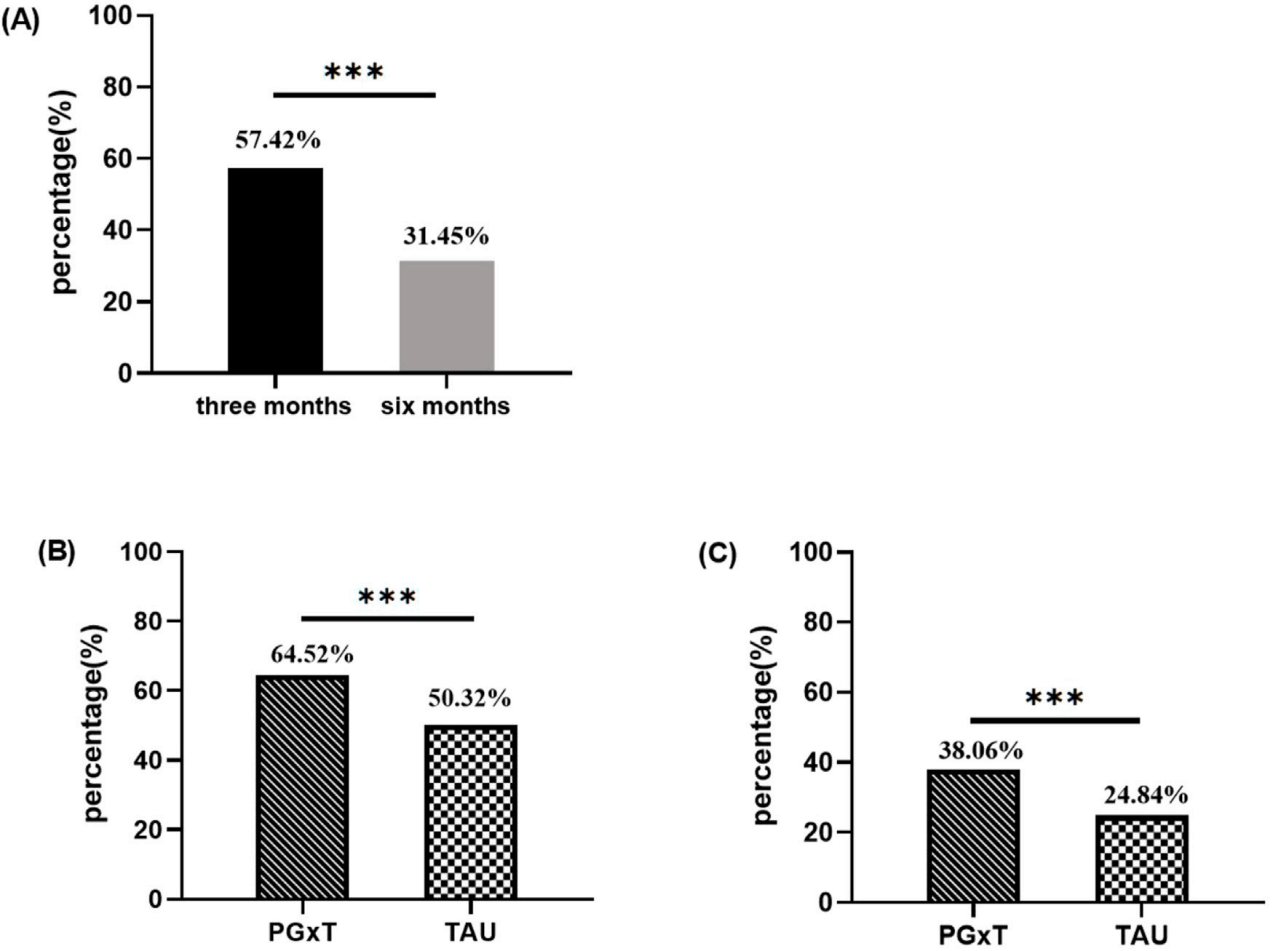
Comparison of medication adherence between PGXT and TAU groups three and six months after hospital discharge. **(A)** Proportion of people with good adherence at 3 and 6 months in the overall population (n = 620). **(B)** Percentage of patients with good compliance at 3 months in both groups. **(C)** Percentage of patients with good compliance at 6 months in both groups. PGXT: pharmacogenomics-guided treatment, TAU: treatment as usual.*p < 0.05, **p < 0.01, ***p < 0.001.

**FIGURE 3:**
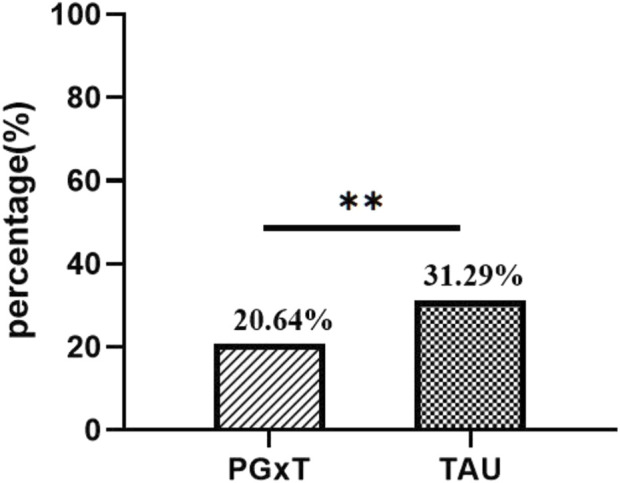
Comparison of ASR in the PGXT group and the TAU group. ASR, antidepressant switching rate ;*p < 0.05, **p < 0.01, ***p < 0.00.

Patients’ medication adherence was compared between the two groups using a primary endpoint analysis. Adherence was higher during the 3-month follow-up period (57.42% of all patients showed good adherence) compared to the 6 months (31.45%), with the difference being statistically significant (*p* < 0.001; Figure 2A). At 3-month follow-up, good adherence rates were 64.52% in the PGxT group and 50.32% in the TAU group (*p* < 0.001; Figure 2B). Similarly, at 6 months, good adherence rates were 38.06% in the PGxT group and 24.84% in the TAU group (*p* < 0.001; Figure 3C). These statistically significant differences in adherence at the three- and 6-month marks indicate that PGxT positively influences long-term medication adherence among patients.

### Antidepressant switching rate

We conducted a comprehensive statistical analysis of the medication patterns for patients in both groups from discharge to the end of the observation period. ASR was significantly higher in the TAU group than in the PGxT group. Specifically, within the PGxT group, 64 patients required adjustments to their antidepressant medications (ASR = 20.64%). In contrast, 97 patients have ASR (31.29%) in the TAU group. This statistically significant difference in ASR between the groups (*p* < 0.01; Figure 3) demonstrates the efficacy of individualized PGxT in significantly reducing the rate of antidepressant switching.

## Discussion

This study substantiates the significant impact of PGxT on adherence to antidepressant regimens. Patients in the PGxT group demonstrated significantly higher adherence during the acute and consolidation phases of treatment than those in the TAU group. PGxT effectively reduced ASR, thus minimizing the trial-and-error approach commonly observed in TAU. These outcomes indicate the substantial value of PGxT in guiding the selection of antidepressant medications, which enhances patient adherence and reduces unnecessary utilization of healthcare resources.

Supporting evidence from prior research further validates these findings. Charland et al. highlighted that participation in KIF6 genetic testing was associated with significantly higher 6-month statin adherence and persistence than matched controls and patients who declined participation (Charland et al., 2013). Most patients with type 2 diabetes or pre-diabetes expressed favorable views toward genetic testing to guide therapy, indicating a substantial increase in their motivation to adhere to medications (Grant et al., 2009). Consistent with our results, an economic analysis of psychiatric pharmacogenomics demonstrated that this approach could decrease direct pharmacy costs, improve adherence, and reduce the use of comorbid medications (Winner et al., 2015).

In a prospective case-control study, Christian et al. found no significant differences in mPDC between patients with and without pharmacogenetic guidance (Christian et al., 2020). However, their study predominantly examined patients with chronic conditions such as diabetes, hypertension, hypercholesterolemia, and chronic heart failure, while our research focused exclusively on patients with depression. Our findings highlight the importance of considering patient differences when selecting antidepressants and dosages to improve treatment adherence.

The annual economic burden of medication non-adherence in the United States is approximately $300 billion (Haga and LaPointe, 2013). Haga et al. (2016) reported that patients expressed support for integrating pharmacogenetic testing into clinical practice to guide treatment decisions and exhibited reduced concerns about medications after such testing. PGxT represents a synthesis of pharmacogenetics and advances in human genomics, allowing exploration of how various genes and their variants contribute to the phenotype of an individual’s drug response. Although numerous randomized controlled trials (RCTs) have affirmed the benefits of multigene PGxT in managing major depressive disorder, outcomes vary. For example, Greden et al. did not observe significant improvement in mean symptom severity with PGxT; however, the approach significantly improved response and remission rates in patients with difficult-to-treat depression compared to standard care (Greden et al., 2019). Pérez et al. (2017) noted that PGxT improved both efficacy and tolerability over TAU in a naturalistic setting. Additionally, Papastergiou et al. found that participants with depressive and generalized anxiety disorders reported greater improvements in depression severity and anxiety, as well as reduced disability for 6 months when receiving PGxT compared to TAU (Papastergiou et al., 2021).

We explored how PGxT affects patient adherence to their antidepressant regimen, an important clinical issue because medication adherence directly affects treatment efficacy and patient health outcomes. By comparing PGxT with TAU in terms of ASR, the study provides insights into how personalised medicine can reduce unnecessary antidepressant switching, which may help to reduce treatment interruptions and associated psychosocial burdens. This study focuses on the use of pharmacogenomics in the treatment of depression, a relatively new area of research, and the results could help to drive the further development of personalised medicine in mental health.

This study has several limitations; firstly, it employed a retrospective design to assess patient medication adherence. While this design facilitates the use of existing data resources, it introduces limitations, particularly in documenting patient comorbidities and controlling potential confounders. For instance, retrospective data collection may lead to information bias due to potential limitations in the completeness and accuracy of medical records. Additionally, selection bias must be considered. To mitigate these limitations, propensity score matching (PSM) was utilized in this study. However, we acknowledge that these measures do not fully eliminate the inherent limitations of retrospective studies. For example, we did not account for patients’ comorbidities, including psychiatric or somatic disorders (e.g., personality disorders, obsessive-compulsive disorder), alcohol use disorders, or cardiovascular disease. Previous studies have shown that patients with these disorders typically exhibit lower medication adherence (Semahegn et al., 2020; Akincigil et al., 2007), and such comorbidities can significantly impact adherence. These conditions may also necessitate complex combination therapies and increase the overall medication burden for patients, potentially leading to a higher likelihood of discontinuing antidepressants compared to medications for somatic disorders (Moritz et al., 2012). Secondly, this study did not stratify patients based on their Hamilton Depression Scale (HAMD) scores and did not assess treatment outcomes for patients due to the scarcity of HAMD scale scores. Thirdly, the data were derived from a single center, limiting the representativeness and generalizability of the findings. Lastly, while this study used retrospective data to demonstrate the impact of PGxT on medication adherence and ASR, it did not employ a randomized controlled trial (RCT) to validate these findings. Future studies should consider a broader range of factors affecting patient adherence and conduct prospective RCTs to compare PGxT with TAU regarding medication adherence.

## Conclusion

Pharmacogenomics-guided treatment significantly improves medication adherence and reduces antidepressant switching rates in patients with major depressive disorder compared to treatment as usual. By integrating genetic insights into treatment decisions, PGxT allows for more personalized and effective therapy, minimizing trial-and-error prescribing. These findings highlight the potential of PGxT to improve clinical outcomes and reduce healthcare resource utilization in the management of depression.

## Data Availability

The data analyzed in this study is subject to the following licenses/restrictions: PDC and ASR related data for the final 620 patients. Requests to access these datasets should be directed to CZ, zhouchunhua@hebmu.edu.cn.
