# Supplementary material for: Effects of pharmacogenomics-guided treatment on medication adherence and the antidepressant switching rate in major depressive disorder
Source: Front Pharmacol. 2024 Nov 29;15:1501381. doi: 10.3389/fphar.2024.1501381 (PMC11639597; doi:10.3389/fphar.2024.1501381)
Supplement: Supplementary file 1 [file DataSheet1.docx]

Supplementary Material

# Supplementary **method**

**Genomic DNA extraction**

We collected 2 mL of peripheral venous blood by EDTA anticoagulation tube, extracted genomic DNA with KingFisher Flex Nucleic Acid Extractor (Thermo Fisher, Waltham, USA), and detected DNA concentration and purity with NanoDrop 2000 (Thermo Fisher, Waltham, USA). The concentration of DNA was required to be controlled at 10 ng/μL above, with the A260/A280 between 1.7 and 2.0.

**SNP genotyping**

The SNP sites were divided into three detection wells for multiple PCR reaction, and the target fragment containing the SNP to be detected was amplified. Reaction system of multiplex PCR (5 μL): 0.5 μL 10x PCR buffer (Agena Bioscience, San Diego, USA), 0.4 μL MgCl_2_ (Agena Bioscience, San Diego, USA), 0.1 μL dNTP mix (Agena Bioscience, San Diego, USA), 0.2 μL PCR enzyme (Agena Bioscience, San Diego, USA), 1.0 μL Primer mix (500 nM each) (Invitrogen, Carlsbad, USA), 0.8 μL nuclease-free water (Invitrogen, Carlsbad, USA), 2 μL genomic DNA. PCR was conducted with Applied Biosystems Veriti 96-Well (Thermo Fisher, Waltham, USA) under the standard conditions: denaturation at 95°C for 2 min, followed by 45 cycles at 95°C for 30 s, annealing temperature 60°C for 60 s, extending 72°C for 60 s and by a final extension at 72°C for 5 min. Then, we added 2 μL SAP reaction mixture after PCR amplification for dephosphorylating and degrading the remaining dNTP. The components of SAP reaction mixture (2 μL): 0.17 μL SAP buffer (Agena Bioscience , San Diego, USA), 0.30 μL SAP enzyme (Agena Bioscience, San Diego, USA), 1.53 μL nuclease-free water (Invitrogen, Carlsbad, USA). SAP reaction was conducted with Applied Biosystems Veriti 96-Well (Thermo Fisher, Waltham, USA) under the standard conditions: 37°C for 40 min, followed by 1 cycle at 85°C for 5 min. After SAP reaction, we added 2 μL extension reaction mixture for single base extension reaction. During the reaction, the extension primer combined with the 5' end sequence of the SNP to be tested and extended one base. The components of extension reaction mixture (2 μL): 0.2 μL iPlex buffer plus (Agena Bioscience, San Diego, USA), 0.2 μL iPlex termination mix (Agena Bioscience, San Diego, USA), 0.04 μL iPlex pro enzyme (Agena Bioscience, San Diego, USA), 0.94 μL extend primers mix (Agena Bioscience, San Diego, USA), 0.62 μL nuclease-free water (Invitrogen, Carlsbad, USA). Extension reaction was also conducted with Applied Biosystems Veriti 96-Well (Thermo Fisher, Waltham, USA) under the standard conditions: 95°C for 30 s, followed by 40 cycles extension (95°C for 5 s, followed by 5 cycle at 52°C for 5 s and 80°C for 5 s) and 72°C for 3 min. The extended product was detected with MassARRAY nucleic acid mass spectrometry system (Agena Bioscience, San Diego, USA), and the results were analyzed with MassARRAY Typer 4.1 software (Agena Bioscience, San Diego, USA).

**CNV detection of *CYP2D6* gene**

The three detection targets (promoter, intron 6, exon 9) of *CYP2D6* gene copy number and the reference gene (*RPPH1* gene) were respectively used for double real time qPCR reaction in the same reaction well, and a sample with known copy number was added as the calibration sample in the experiment. The components of real time qPCR reaction (20 μL): 10 μL 2x TaqMan genotyping master mix (Agena Bioscience, San Diego, USA), 1 μL 20x TaqMan copy number assay (Agena Bioscience, San Diego, USA), 1 μL 20x TaqMan copy number reference assay (Agena Bioscience, San Diego, USA), 6 μL nuclease-free water (Invitrogen, Carlsbad, USA), 2 μL genomic DNA. Real time qPCR reaction was conducted with Applied Biosystems 7500 (Thermo Fisher, Waltham, USA) under the standard conditions: 50°C for 2 min, 95°C for 10 min, followed by 40 cycles at 95°C for 15 s and 60°C for 60 s. Then, we used CopyCaller V2.1 software (Thermo Fisher, Waltham, USA) calculate the copy number of these three targets by ΔΔ Ct relative quantitative method.

**Detection of 5-HTTLPR polymorphism of *SLC6A4* gene**

We used PCR reaction to amplify the 5-HTTLPR region located in the promoter region of *SLC6A4* gene. Reaction system of PCR (25 μL): 2.5 μL expand long template buffer with Mg^2+^ (Roche, Basel, Switzerland), 1 μL PCR nucleotide mix (Roche, Basel, Switzerland), 1 μL enzyme mix (Roche, Basel, Switzerland), 1 μL 5-HTTLPR forward primer (Invitrogen, Carlsbad, USA), 1 μL 5-HTTLPR reverse primer (Invitrogen, Carlsbad, USA), 18.5 μL nuclease-free water (Invitrogen, Carlsbad, USA). PCR was conducted with Applied Biosystems Veriti 96-Well (Thermo Fisher, Waltham, USA) under the standard conditions: denaturation at 94°C for 2 min, followed by 30 cycles at 94°C for 30 s, annealing temperature 58°C for 30 s, extending 72°C for 60 s and by a final extension at 72°C for 10 min. Then, we used Agilent 2100 Bioanalyzer system (Agilent, Palo Alto, USA) to analyze the amplification products by capillary electrophoresis, and judged the genotype of the sample to be tested with the quality control sample of S/L genotype as the scale.

# Supplementary Figures and Tables

## Supplementary Figures


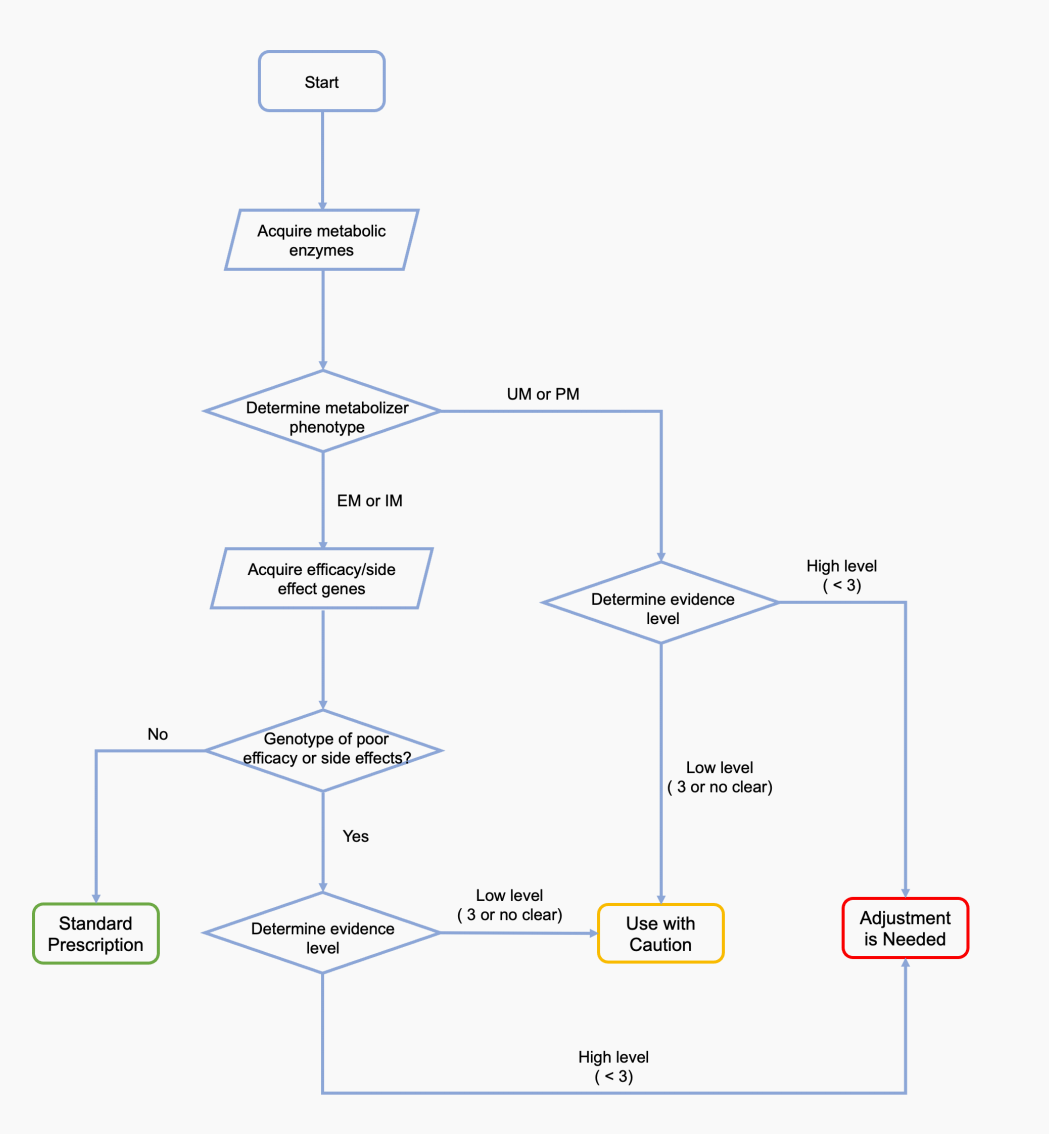


- **Supplementary Figure S1.** Principles of interpretation of drug recommendations
-
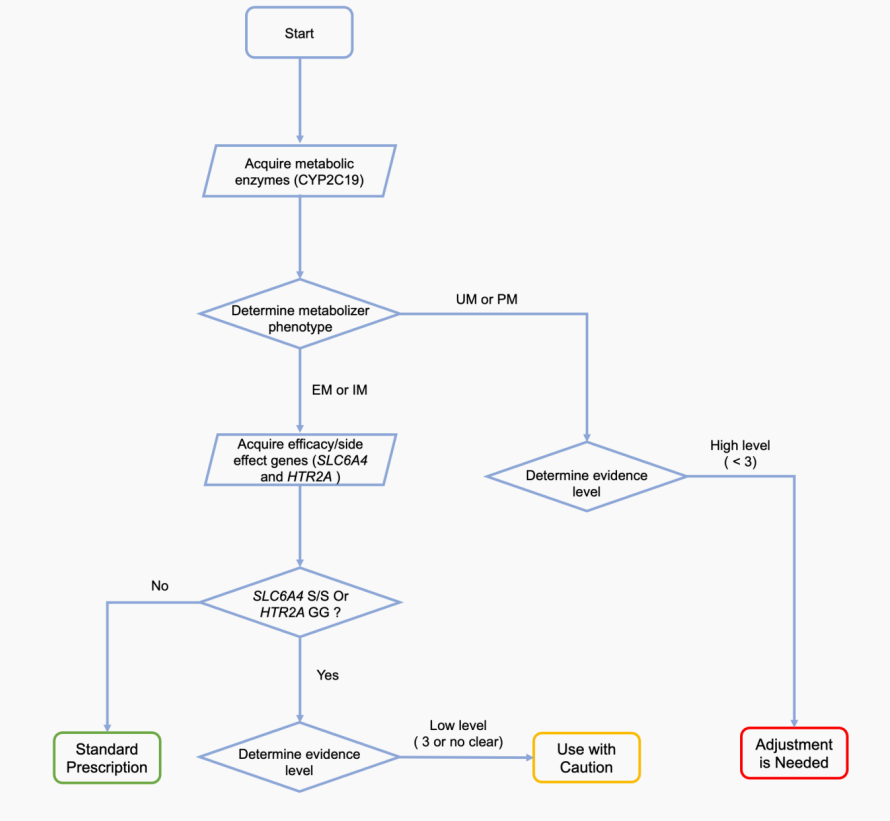


**Supplementary Figure S2.**Process of medication recommendation based on PGxT using escitalopram as an example. CYP2C19 UM/PM: “adjustment is needed”; CYP2C19 EM/IM + (SLC6A4 SS or HTR2A GG): “use with caution”; CYP2C19 EM/IM + (SLC6A4 L/L or L/S ) + HTR2A CC/CG: “standard prescription.”

# 2.2 Supplementary Tables

**Supplementary Table S1.**The 33 loci of 8 genes associated with depression in this study

| Genes | Loci | Allele |
| --- | --- | --- |
| *CYP2D6* | *rs1065852* | *10 |
|  | *rs1135822* | *49 |
|  | *rs1135840* | *14 |
|  | *rs16947* | *14 |
|  | *rs28371725* | *41、*69、*119 |
|  | *rs35742686* | *3 |
|  | *rs3892097* | *4 |
|  | *rs5030655* | *6 |
|  | *rs5030865* | *14 |
|  | *rs72549349* | *44 |
|  | *rs72549352* | *21 |
| *CYP2C19* | *rs12248560* | *17 |
|  | *rs12769205* | *2、*35 |
|  | *rs28399504* | *4 |
|  | *rs3758581* | *2、*3、*17、*4、*6、*35 |
|  | *rs4244285* | *2 |
|  | *rs4986893* | *3 |
|  | *rs72552267* | *6 |
| *CYP2B6* | *rs2279343* | *4、*6、*7、*26、*34、*36 |
|  | *rs28399499* | *18 |
|  | *rs3211371* | *5、*7、*33、*34 |
|  | *rs34223104* | *22、*34、*36 |
|  | *rs3745274* | *6、*7、*9、*26、*34、*36 |
|  | *rs8192709* | *2 |
| *CYP1A2* | *rs2069514* | / |
|  | *rs762551* | / |
| *ABCB1* | *rs1045642* | / |
|  | *rs2032583* | / |
| *HTR1A* | *rs10042486* | / |
|  | *rs6295* | / |
| *HTR2A* | *rs2770296* | / |
|  | *rs9316233* | / |
| *SLC6A4* | *5-HTTLPR* | / |

**Supplementary Table S2.** Metabolism-related genes with antidepressant drug therapy.

| **CYP Enzyme** | **Antidepressants** | **Phenotype** | **Definition** | **Example** |
| --- | --- | --- | --- | --- |
| CYP2D6 (Hicks *et al.,*2015) | Fluvoxamine, Paroxetine | UM | Individuals carrying functional allele duplication | *1/*1xN, *1/*2xN, *2/*2xN |
|  |  | NM | Individuals carrying two normal alleles or two reduced alleles or one normal and one non-functional allele or one normal and one reduced allele | *1/*1, *1/*2, *1/*4, *1/*5, *1/*9, *1/*41, *2/*2, *41/*41 |
|  |  | IM | Individuals carrying a functional decline and a non-functional allele | *4/*10, *4/*41, *5/*9 |
|  |  | PM | Individuals carrying only non-functional alleles | *3/*4, *4/*4, *5/*5, *5/*6 |
| CYP2C19 (Hicks *et al.,*2015) | Citalopram, Escitalopram | UM | Individuals carrying two functional increase alleles or one normal functional allele and one functional increase allele | *17/*17, *1/*17 |
|  |  | NM | Individuals carrying two functional alleles | *1/*1 |
|  |  | IM | Individuals carrying a normal allele or an increased allele and a non-functional allele | *1/*2, *1/*3,*2/*17 |
|  |  | PM | Individuals carrying two non-functional alleles | *2/*2,*2/*3,*3/*3 |
| CYP2B6 (Benowitz *et al.,*2013) | Bupropion, Sertraline | UM | Individuals carrying two functional increase alleles | *4/*4,*22/*22,*4/*22 |
|  |  | RM | Individuals carrying one normal allele and an increased allele | *1/4,*1/22 |
|  |  | NM | Individuals carrying two normal functional alleles | *1/1 |
|  |  | IM | Individuals carrying a normal allele and a reduced allele or a normal allele and a non-functional allele or a functional increase allele and a reduced allele or a functional increase allele and a non-functional allele | *6/6,*18/18,*6/18 |
|  |  | PM | Individuals carrying two functional decreased alleles or two nonfunctional alleles or one functional decreased allele and one nonfunctional allele | *6/6,*18/18,*6/18 |

**Supplementary Table S3.** Efficacy-related genes associated with antidepressant drug therapy.

| Gene | Allele | Association | Antidepressants |
| --- | --- | --- | --- |
| CYP1A2 | rs762551 allele A | Associated with an increased risk of fatigue | Paroxetine (Lin *et al.,*2010) |
|  | *1C (rs2069514) | Associated with decreased enzyme activity | Argomepratine (Saiz-Rodríguez *et al.,*2019) |
| ABCB1 | rs1045642 AA or AG genotype | Associated with lower response to SSRIs | SSRIs (Jeleń *et al.,*2015) |
|  | rs2032583 AG or GG genotype | Associated with an increased likelihood of clinical remission and an increased risk of adverse effects | (Uhr *et al.,*2008) |
| HTR1A | rs6295 GG genotype in Asian populations | Associated with an increased response to antidepressants compared to patients with the CG and CC genotypes |  |
|  | rs10042486 CC genotype | Associated with an increased response to milnacipran, fluvoxamine and paroxetine compared to patients with the CT or TT genes | Milnacipran, Fluvoxamine, Paroxetine (Kato *et al.,*2009) |
| HTR2A | rs2770296 G allele homozygous | Associated with a high probability of clinical remission when treated with bupropion | Bupropion (Tiwari *et al.,*2013) |
|  | rs9316233 allele C | Associated with an increased antidepressant response | (Uher *et al.,*2009) |
| SLC6A4 | 5-HTTLPR S allele | Associated with a reduced response to fluvoxamine and an increased risk of side effects | Fluvoxamine (Zanardi *et al.,*2001;Popp *et al.,*2006) |

- **Supplementary Table S4.** Phenotype and evidence levels of the 14 drugs/8 genes.

| Drugs | Genes | Types | Evidence levels |
| --- | --- | --- | --- |
| Escitalopram | *CYP2C19* | Metabolism | 1A |
|  | *SLC6A4* | Effectiveness | 4 |
|  | *HTR2A* | Effectiveness | 3 |
| Sertraline | *CYP2C19* | Metabolism | 1A |
|  | *SLC6A4* | Effectiveness | 4 |
| Citalopram | *CYP2C19* | Metabolism | 1A |
|  | *SLC6A4* | Effectiveness | 4 |
| Fluoxetine | *CYP2D6* | Metabolism | 3 |
|  | *HTR1A* | Effectiveness | 3 |
| Paroxetine | *CYP2D6* | Metabolism | 1A |
|  | *HTR1A* | Effectiveness | 3 |
| Fluvoxamine | *CYP2D6* | Metabolism | 1A |
|  | *SLC6A4* | Effectiveness | 3 |
| Venlafaxine | *CYP2D6* | Metabolism | 2A |
|  | *ABCB1* | Effectiveness and side effect | 3 |
| Duloxetine | *CYP2D6* | Metabolism | 2A |
|  | *CYP1A2* | Metabolism | 3 |
| Milnacipran | *HTR1A* | Effectiveness | 3 |
|  | *HTR1A* | Effectiveness | 3 |
| Vortioxetine | *CYP2D6* | Metabolism | 1A |
| Mirtazapine | *CYP2D6* | Metabolism | 2A |
|  | *SLC6A4* | Side effect | 3 |
| Agomelatine | *CYP1A2* | Metabolism | Literature support |
|  | *ABCB1* | Effectiveness | 3 |
| Bupropion | *CYP2B6* | Metabolism | 2A |
|  | *HTR2A* | Effectiveness | 3 |
| Mianserin | *CYP2D6* | Metabolism | 3 |
